# Mitigation of Physical Aging of Polymeric Membrane Materials for Gas Separation: A Review

**DOI:** 10.3390/membranes13050519

**Published:** 2023-05-17

**Authors:** Danila S. Bakhtin, Stepan E. Sokolov, Ilya L. Borisov, Vladimir V. Volkov, Alexey V. Volkov, Vadim O. Samoilov

**Affiliations:** 1 A.V. Topchiev Institute of Petrochemical Synthesis, Russian Academy of Sciences, 119991 Moscow, Russia; db2@ips.ac.ru (D.S.B.); sokolovste@ips.ac.ru (S.E.S.); vvvolkov@ips.ac.ru (V.V.V.); samoilov@ips.ac.ru (V.O.S.); 2 Biological and Environmental Science, and Engineering Division (BESE), Advanced Membranes and Porous Materials Center (AMPM), King Abdullah University of Science and Technology, Thuwal 23955, Saudi Arabia

**Keywords:** membrane gas separation, glassy polymers, physical aging, mitigating aging, thin film composite membranes, mixed-matrix membranes

## Abstract

The first commercial hollow fiber and flat sheet gas separation membranes were produced in the late 1970s from the glassy polymers polysulfone and poly(vinyltrimethyl silane), respectively, and the first industrial application was hydrogen recovery from ammonia purge gas in the ammonia synthesis loop. Membranes based on glassy polymers (polysulfone, cellulose acetate, polyimides, substituted polycarbonate, and poly(phenylene oxide)) are currently used in various industrial processes, such as hydrogen purification, nitrogen production, and natural gas treatment. However, the glassy polymers are in a non-equilibrium state; therefore, these polymers undergo a process of physical aging, which is accompanied by the spontaneous reduction of free volume and gas permeability over time. The high free volume glassy polymers, such as poly(1-trimethylgermyl-1-propyne), polymers of intrinsic microporosity PIMs, and fluoropolymers Teflon^®^ AF and Hyflon^®^ AD, undergo significant physical aging. Herein, we outline the latest progress in the field of increasing durability and mitigating the physical aging of glassy polymer membrane materials and thin-film composite membranes for gas separation. Special attention is paid to such approaches as the addition of porous nanoparticles (via mixed matrix membranes), polymer crosslinking, and a combination of crosslinking and addition of nanoparticles.

## 1. Introduction

Membrane gas separation processes are widely used today in many large-scale separation processes [1]. The first commercial application of hollow fiber separation membranes was hydrogen recovery from ammonia purge gas in the ammonia synthesis loop. Prism^®^ membranes, marketed by Monsanto (Permea) in the late 1970s were the first commercialized polysulfone hollow fiber membranes for this purpose. Since the mid-1990s, Permea has been a division of Air Products. In the late 1970s, Kuskovo Chemical Plant (Moscow, Russia) started production of a flat asymmetric membrane PVTMS [2]. Cryogenmash (Balashikha, Russia) produced PVTMS plate and frame modules, as well as gas separation plants for hydrogen recovery and oxygen/nitrogen separation. The PVTMS membrane was produced until the mid-1990s. To date, Prism^®^ hydrogen separation membranes have also been used for syngas ratio adjustment and in the recovery of hydrogen in refinery hydrotreaters. It is important to emphasize that the global annual demand for hydrogen is estimated at 115 million tons, and this value is expected to increase by almost six-fold by 2050 [3].

Industrially, membranes are currently used for acid gas removal (natural gas sweetening), nitrogen generation from the air, ammonia purge gas recovery, refinery gas purification and dehydration (Table 1). Processing of natural gas is, by far, the largest industrial gas separation application, first of all, for the removal of carbon dioxide from natural gas (CO_2_/CH_2_) [4]. In addition, the separation of nitrogen from the air (nitrogen generation) represents about half of the current gas separation market [1].

**Table 1:** Primary current industrial gas separation applications for polymer membranes (adopted from [5]).

Gas Pair	Application
CO_2_/CH_4_	Acid gas treatment
	Biogas separation
N_2_/O_2_	Nitrogen generation from the air
H_2_/N_2_	Ammonia purge gas recovery
H_2_/CH_4_	Refinery gas purification
H_2_/CO	Syngas ratio adjustment
H_2_O/Air	Dehydration

**Figure 1:**
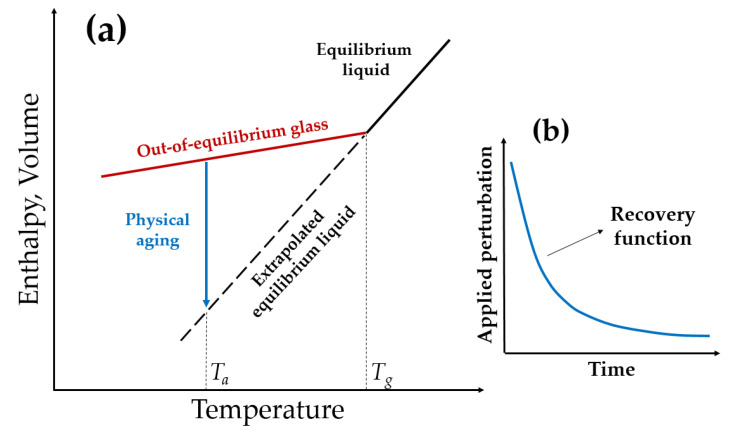
Schematic representation of the temperature dependence of volume or enthalpy of glass-forming materials (**a**); Schematization of the kinetics of volume or enthalpy recovery (**b**) (adopted from [7]).

As can be seen from Table 2, glassy polymers (polysulfone, cellulose acetate, polyimide, polycarbonate, poly(phenylene oxide), and polyvinyltrimethylsilane) are the most commonly used gas separation membranes due to their excellent combination of permeability–selectivity properties and processability into high flux membranes. It is important to notice that the same polymers (polysulfone, polyimides, cellulose acetate, substituted polycarbonate, and poly(phenylene oxide)) have been used for more than 30 years. On the other hand, glassy polymers undergo a process termed physical aging, because polymer glasses are thermodynamically in non-equilibrium. When a polymeric solution is cooled at constant pressure from an equilibrium liquid, the transformation into glass occurs at a temperature T_g_, when the macromolecular rearrangements necessary for the material to adapt to changing temperature slow down to such an extent that it takes an order of magnitude longer time than that available in the observed conditions. Since the coefficient of thermal expansion undergoes a sharp change at T_g_, in the glassy state of a polymer, there is always a difference between the volume of the non-equilibrium glassy material and the volume extrapolated from equilibrium. Therefore, glassy polymer materials slowly change from a non-equilibrium state to a thermodynamically equilibrial one over time. A schematic representation of the temperature dependence of volume or enthalpy of glass-forming materials as well as the kinetics of volume or enthalpy recovery is shown in Figure 1. Thus, the thermodynamically stable state tends to be slowly recovered during the course of physical aging (Figure 1b). As a result of this phenomenon, the density of the polymer decreases, thus reducing the free volume and gas transport properties over time.

**Table 2:** Important commercially available membranes for gas separation (adopted from [6]).

Membrane	Supplier	Material	Gas Separation	Module Type
Prism	Air Products/Permea	Polysulfone	H_2_/CO	Hollow fiber
			H_2_/N_2_	
			H_2_/CH_4_	
			O_2_/N_2_	
Cynara	Cameron	Cellulose acetate	CO_2_/CH_4_	Hollow fiber
Medal	Air Liquide	Polyimide/polyaramide	CO_2_/CH_4_	Hollow fiber
			N_2_/CH_4_	
			H_2_S/CH_4_	
Separex	UOP	Cellulose acetate	CO_2_/CH_4_	Spiral wound
			H_2_O/CH_4_	
Grace	UOP	Cellulose acetate	CO_2_/CH_4_	Spiral wound
			H_2_O/CH_4_	
Generon	IGS, Inc.	Polycarbonate, incl tetrabromo	O_2_/N_2_,	Hollow fiber
			H_2_/N_2_,	
			H_2_/CH_4_	
IMS	Praxair	Polyimide	O_2_/N_2_	Hollow fiber
			(N_2_ generation)	
UBE	Ube Industries	Polyimide	H_2_O/Air	Hollow fiber
			O_2_/N_2_	
			(N_2_ generation)	
			CO_2_/CH_4_	
Parker	Parker Hannifin	poly(phenylene oxide)	O_2_/N_2_	Hollow fiber
			(N_2_ generation)	
PVTMS ^1^	Cryogenmash	Polyvinyltrimethylsilane	H_2_/N_2_	Plate and frame
			O_2_/N_2_	

^1^ Flat asymmetric membrane PVTMS was produced by Kuskovo Chemical Plant (Moscow, Russia) from the late 1970s until the mid-1990s.

Until the 1970s, glassy polymers were considered materials of low permeability and high gas separation selectivity. Poly(vinyltrimethyl silane) (PVTMS), having a nitrogen permeability coefficient (10 Barrer) forty times higher than for PSF, turned out to be the first instance of a high permeability glassy polymer [2]. The next achievement was associated with the extra high permeability glassy polymer, poly(1-trimethylsilyl-propyne) (PTMSP), first synthesized at Kyoto University in 1983 [8]. The unique structure of the free volume of PTMSP and other highly permeable substituted polyacetylenes was attributed to the presence of a rigid polyene chain with bulky side substituents. The Kuhn segments of substituted polyacetylenes are 35–100 Å [9] (15–40 units per segment), while for PVTMS, a polymer with a vinyl chain and the same bulky side trimethylsilyl substituent, the Kuhn segment does not exceed 15 Å (six units) [10]. The first estimates of the free volume of PTMSP showed that this polymer is characterized by an extremely high value of non-equilibrium (non-relaxed) free volume fraction, 20–26%, consisting of interconnected “voids” (free volume elements) with the narrowest “chain-to-chain” distance of 0.3–0.5 nm [11,12]. The results of molecular modeling and positron annihilation data indicate that PTMSP has a bimodal pore size distribution with peaks in the region of 0.4 nm and 0.6–0.8 nm [13,14].

**Figure 2:**
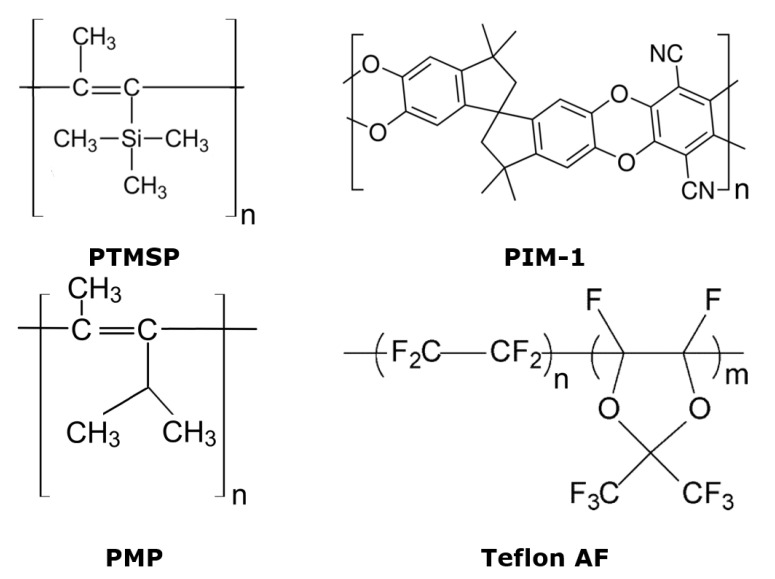
Chemical structure of glassy polymers, mentioned in this work: poly(1-trimethylsilyl-propyne), poly(4-methyl-2-pentyne), polymer of intrinsic microporosity PIM-1, and fluoropolymer Teflon^®^ AF.

Today, the group of highly permeable glassy polymers also includes PIMs [15], poly(4-methyl-2-pentyne) [16], poly(1-trimethylgermyl-1-propyne) [17] polynorbornens [18], poly(tricyclononenes) [19], the fluoropolymers Teflon^®^ AF and Hyflon^®^ AD [20,21,22], and some others. Over the past few decades, several methods have been proposed to mitigate the physical aging of glassy polymers and improve the durability of membranes based on them. Herein, we review these techniques, and well-known membrane glassy materials (listed in Figure 2) were used as examples. Special attention in this paper is given to the physical aging of thin-film composite membranes that have more practical application for separation processes than the dense films.

## 2. Physical Aging

### 2.1. Physical Aging of Glassy Polymers

Amorphous polymers are structurally inhomogeneous materials with sizes of inhomogeneity ranging from units to tens of angstroms. Numerous studies explain the features of physical aging processes using assumptions about the inhomogeneous structure of polymer glasses, particularly those about the existence of ordered domains in them [23,24].

Paul and coworkers [25,26,27,28,29,30,31,32,33,34,35,36] made the most significant contribution to the study of the physical aging of polymeric membranes based on glassy polymers. A number of reviews are directly devoted to the physical aging problem [1,15,37,38,39,40,41,42]. Based on the free volume and chain mobility concept, several mechanisms such as lattice contraction and diffusion of free volume from the polymer matrix have been proposed to describe physical aging [37,43].

### 2.2. Physical Aging of Dense Membranes

Yampolskii et al. [44] studied the change in the free volume in PTMSP by positron annihilation lifetime spectroscopy at different temperatures: 25, 100, and 128 °C. The decrease in free volume with the aging time was interpreted as a decrease in the number of free volume elements during aging, which is in good agreement with the free volume model proposed by Struik in 1978 [45].

McCaig and Paul [26] studied the permeability of oxygen through polyarylate films of different thicknesses. It was found that the oxygen permeability decreases with aging time, and rather complex patterns of changes in gas permeability with aging time are observed for films of different thicknesses. The authors distinguish two ranges of polyarylate film thicknesses. For thick films (from several microns to several tens of microns), the loss of permeability due to aging is relatively small, and the aging rate does not depend on the film thickness. For thinner films (from tenths to several microns), the loss of oxygen permeability due to aging is more significant. In this case, the aging rate depends on the film thickness: the thinner the film, the stronger the effect of reducing the permeability. According to [26], one of the possible explanations for accelerated permeability loss in thin polymer films is free volume diffusion. During the polymer aging, non-equilibrium free-volume elements («holes») diffuse from the sample bulk to its surface and the thinner the film thickness, the faster its volume change. The second mechanism—the theory of “lattice contraction”—assumes that the decrease in intermolecular distances during aging occurs simultaneously throughout the entire volume of the sample. The entire set of experimental data on the aging of thick and thin polyarylate films can be consistently explained if it is assumed that both free volume relaxation mechanisms occur simultaneously.

### 2.3. Physical Aging of Thin-Film Membranes

Pfromm and Koros [46] showed that the rate of aging of glassy polymer films can depend on their initial thickness. The authors studied the aging of films made of 6FDA-IPDA polyimide and polysulfone with the same thermal history. It was shown that the aging of “thin” films (0.5 μm) occurs much faster than that of intermediate (2.5 μm) and thick (~25 μm) films. Thus, 6000 h after quenching, the ideal He/N_2_ selectivity of 6FDA-IPDA thin films increased by more than one and a half times (from 45 to 70), while for other samples, the increase in selectivity did not exceed 15%. A similar aging behavior was also found for polysulfone.

At the same time, Dorkenoo and Pfromm [47] believed that the accelerated aging of thin films (thickness less than 1 µm) can be qualitatively explained based on well-known concepts, according to which the glass transition temperature of thin films (thickness less than 1 µm) decreases markedly with a decrease in their thickness [48,49]. Other things being equal, this circumstance increases the intensity of molecular motion in thin films, which determines the increase in the rate of physical aging in them.

Huang and Paul [25], using ellipsometry, studied the change in the refractive index of thin films of three glassy polymers—polysulfone, polyimide, and poly(2,6-dimethyl-1,4-phenylene oxide). These thin films less than 1 µm thick were kept up to 6000 h at 35 °C. A pronounced aging effect, monitored by a change in the refractive index, was observed because of the densification of glassy polymers.

It is of interest to compare the results of two studies on the gas permeability of Matrimid^®^ films of various thicknesses by Paul and coworkers [27,50]. In both studies, films with different thicknesses were obtained by spin casting on a silicon wafer: 0.39–22.4 µm [50] and 18–550 nm [27]. In the case of ultrathin Matrimid^®^ films, a thin layer of rubbery PDMS was coated directly on top of the Matrimid^®^ film by spin casting of its solution in cyclohexane. All films were heated above the polymer’s T_g_ value in the free-standing state to allow relaxation of any stresses and then quenched to room temperature.

**Figure 3:**
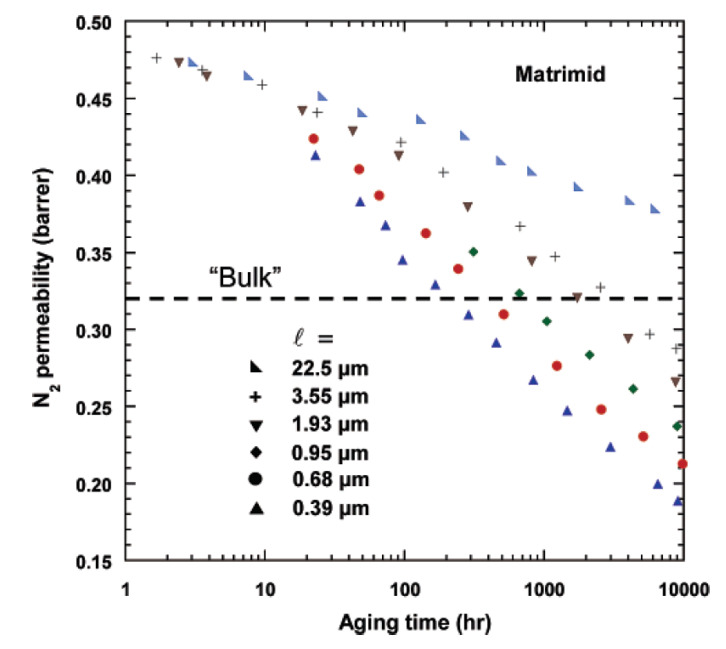
Nitrogen permeability coefficients of Matrimid^®^ films of various thicknesses, as a function of aging time at 35 °C. Reprinted with permission from [50]. Copyright 2007 American Chemical Society.

**Figure 4:**
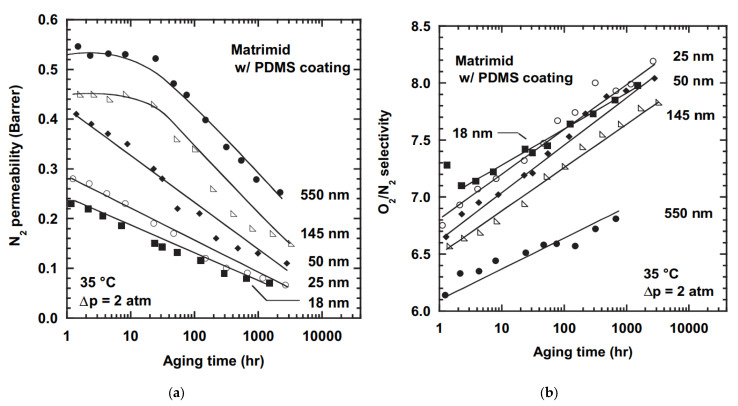
Influence of physical aging on nitrogen permeability (**a**) and O_2_/N_2_ ideal selectivity (**b**) in Matrimid^®^/PDMS films of various thicknesses, as a function of aging time at 35 °C. Lines were generated from the modified Struik model (adopted from [27]).

Gas transport properties of Matrimid films of various thicknesses as a function of aging time at 35 °C are presented in Figure 3 and Figure 4 for Matrimid and Matrimid/PDMS, respectively. It can be seen that the decrease in membrane thickness led to more pronounced physical aging. After 1000 h of aging, the nitrogen permeability of the 25 nm film dropped to a record 0.05 Barrer, while the ideal O_2_/N_2_ selectivity increased by 25% during this time. In the case of a film 22.5 µm thick, the gas permeability coefficient after the same 1000 h of aging turned out to be six times higher (0.38 Barrer). It is important to note that the nitrogen gas permeability coefficients turned out to be similar in different experiments for films of similar thicknesses (0.39 and 0.55 µm). So, after 1000 h of aging, they were 0.27 and 0.28 Barrer, respectively, despite the difference in synthesis and characterization protocols. Huang and Paul [50] explained the accelerated aging of thin films through the increased mobility of polymer chains at the “polymer film/substrate” interface compared to their mobility in the “bulk” of the polymer in the case of thick films (more than 10 μm).

An increase in the mobility of polymer chains should lead to a more equilibrial packing of chains and, consequently, to a higher packing density. The experimental determination of the density of films of various thicknesses [51,52] confirms this point of view.

## 3. Mitigating Physical Aging

Several techniques are currently being explored to mitigate the physical aging problem in high free volume glassy polymers [15] such as (i) variation of polymer backbone design and architecture, (ii) polymer post-synthetic modification, (iii) post-modification of the finished membranes, (iv) the formation of copolymers and blending of polymers (v) the addition of non-porous nanoparticles, and (vi) the addition of microporous nanomaterials.

In this regard, several approaches, such as the addition of non-porous and porous materials [1,15,39,40,53,54,55,56,57,58,59,60], post-modification of the prepared membranes via polymer crosslinking [30,61,62], a combination of crosslinking and addition of nanoparticles [63,64,65,66,67], and changes in the polymer backbone design, have been proven to be controlling in aging in high free volume glassy polymers. In this section, we separately discuss methods of mitigating physical aging for freestanding polymer films and thin-film composite membranes. As we mentioned earlier, aging strongly depends on the thickness of the polymer film. A different aging behavior can be expected in the case of thin polymer film deposited on the porous support due to the lower mobility of polymeric chains situated near the interface with the support (so-called “anchoring” effect).

### 3.1. Dense Membranes

In works [30,61,63,68,69], it was shown that the crosslinking of PTMSP and PMP with bis-azides enabled a decrease in the effect of physical aging in terms of the decline of gas permeability compared with neat polymeric films. Another approach to obtaining a cross-linked system based on high free volume glassy polymers is to form an interpenetrating polymer network, which is a mixture of linear macromolecules of the main (matrix) polymer with a network of chains of another cross-linkable polymer. Fritsch et al. [70] suggested restricting the swelling of PIM-1 or PIM in organic solvents by forming an interpenetrating polymer network by the crosslinking of polyethyleneimine (PEI) introduced in the glassy polymer by using poly(ethylene glycol) diglycidyl ether (PEGDE). The obtained cross-linked PIM-1/PEI membranes were stabilized in n-heptane, toluene, chloroform, tetrahydrofuran, and alcohols and were successfully utilized for organic solvent nanofiltration. Those tested in organic solvent nanofiltration showed better retention performance and MWCO compared to the industrial OSN membrane Starmem^TM^ 240. Bazhenov et al. [62] used the semi-interpenetrating polymer network approach to obtain membranes based on PTMSP. Dense membranes (films) of interpenetrating polymer network of PTMSP with crosslinked PEI/PEGDE were prepared by casting the 0.5 wt% solutions of PTMSP/PEI blend in chloroform onto a cellophane support at room temperature. The freshly prepared films were immersed in 4 wt% solution of the crosslinking agent PEGDE in methanol in order to crosslink the PEI amino groups. Several dense membranes (30 µm) with different content of PEI (0, 4, 10, 20 wt%) were prepared. Based on gas transport properties (CO_2_, N_2_) and swelling of dense membranes PTMSP/PEI in chloroform, the optimal concentration of PEI was selected as 4%.

Hill and coworkers [39,55,56,57,71,72] made an important breakthrough in the mitigation of physical aging in high permeable glassy polymers by using a new type of porous filler, porous aromatic frameworks (PAF), proposed earlier as highly porous material [73]. The introduction of PAF into highly permeable glassy polymers improved their gas permeability characteristics and markedly suppressed physical aging due to reduced macrochain mobility as a result of the partial intrusion of polymer segments into porous fillers. In particular, it was demonstrated that dense films (100–150 µm thick) based on PTMSP, PMP, or PIM-1 that contained 10 wt% of PAF revealed only 5–7% of the reduction in CO_2_ permeability over about 240 days, while non-filled PTMSP, PMP, and PIM-1 exhibited a more pronounced decrease in the gas transport on the level of 38–62% [55]. Another advantage of PAF fillers was attributed to their highly porous nature, which enabled a noticeable increase in gas permeability. For instance, after one year of storage, the dense PTMSP film loaded with 10 wt% of PAF-1-Li_6_C_60_ demonstrated CO_2_ permeability of 50,600 Barrer, whereas unfilled PTMSP films showed a drop in permeability from 29,800 to 13,600 Barrer during the same period of time [56].

Hill and coworkers [56,74] demonstrated that the PTMSP side chain can partially penetrate the pores of PAF-1, resulting in the rigidity of the overall polymer structure and preventing physical aging in the membranes. This process was recently termed porosity-induced side chain adsorption (PISA) [75]. One can assume that applying additives with a significantly larger pore volume and surface area can improve the effect of side chain intrusion into the additive’s porous structure, resulting in stable membrane performance with time. Furthermore, as mentioned by the authors of [75,76], PAF-like materials can be considered promising additives toward the reduction of physical aging in polymers of intrinsic microporosity, but the synthesis of such materials is quite complex and hinders commercial implementation. Therefore, investigations were conducted on the addition of hypercrosslinked polymers into PTMSP: poly(dichloroxylene) (p-DCX) [74,75], hypercrosslinked polystyrene (HCL-PS) [76], and IR-pyrolyzed PAN [60].

**Figure 5:**
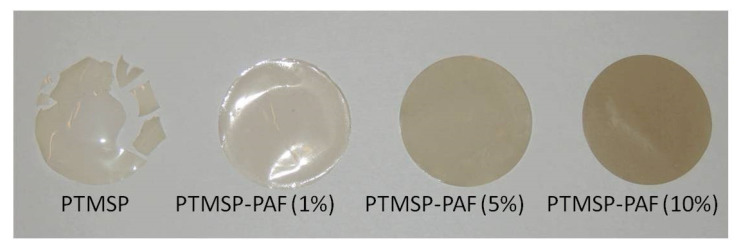
Samples of PTMSP dense membranes with PAF-11 additives after 510 h of annealing at 100 °C. Reprinted from [77] with permission from Elsevier.

Volkov et al. [77] studied stabilization of the gas transport properties of PTMSP by using 30–40 µm dense films loaded with PAF-11 fillers. The time-temperature superposition was successfully utilized to substantially shorten the monitoring time from about one year to several hundred hours by annealing PTMSP membranes at 100 °C. As a result, samples of PTMSP with different contents of PAF-11 were annealed in air at 100 °C for 510 h compared to 240–365 days at room temperature [39,55,56,57]. The sample of neat PTMSP showed a lack of mechanical resistance and was fractionated after 200 h of annealing (Figure 5). Thus, the addition of PAF-11 increased the resistance of the PTMSP/PAF-11 hybrid materials to mechanical stresses and reduced the rate of relaxation processes. Gas transport characteristics of the PTMSP sample containing 10 wt% of PAF-11 became stable upon annealing within the short time interval (100–200 h at 100 °C). However, for all other samples containing 5 wt% of PAF-11 or less, gas permeability gradually decreased with time [77].

In [57], metal-organic polyhedra (MOP) with differing lengths and chemical nature of their side groups were proposed to mitigate the physical aging of glassy polymers. It was shown that the aging of the resulting mixed-matrix membranes made of PTMSP/MOP was significantly reduced due to the non-covalent molecular interlocking of the polymer chains. The best result was obtained for 20 wt% addition of MOP with tert-butyl substituent (tBu-MOP): over 365 days of aging, the CO_2_ permeability decreased by only 20%, while for pure PTMSP this value was 73%.

In many studies, the chemical crosslinking of the polymer matrix was considered an alternative to the addition of nanoparticles of various nature to mitigate physical aging [60,63,64,65,69,78,79]. Hägg and coworkers [63,69] investigated the possibility of stabilizing the gas transport properties of PTMSP and PMP by crosslinking polymers with bis-azides and the simultaneous addition of nonporous SiO_2_ (fumed silica) and TiO_2_ nanoparticles. For both PTMSP and PMP, the gas permeability stability of cross-linked polymer and cross-linked polymer/filler membranes was improved. The physical aging of PMP/silica nanocomposite membrane was also reported by Merkel et al. [80]. The gas permeability of nanocomposites was still unstable over time. Thus, as demonstrated with PMP [69], the crosslinking of polymer chains and a combination of crosslinking with fumed silica additives appeared to be a more efficient approach for mitigating physical aging in high free volume glassy polymer materials.

Soaking in “poor” solvents such as methanol or ethanol can be considered an approach to partially restore the gas transport properties of glassy polymers due to the swelling of the polymer matrix followed by the desorption of solvent molecules [44,81,82,83]. However, this technique cannot mitigate physical aging.

Pinnau and coworkers [84,85,86] tried to tune the molecular design of membrane polymers and synthesized a series of rigid ladder-type diamines from readily available bromoanilines and norbornadiene in one step using facile catalytic arene−norbornene annulation (CANAL). These membranes demonstrated high permeability, moderate selectivities of industrially important gases, and relatively slow aging. At the moment, experiments to study the aging of CANAL membranes are limited to dense membranes from 50 µm thick with a duration up to six months.

### 3.2. Thin Film Composite Membranes

It is well known that thickness plays an important role in the physical aging of glassy polymers, and thinner films undergo more pronounced aging. However, industrial gas separation membranes consist of a very thin, dense selective layer (submicron level), and, therefore, the study of physical aging of thin films is very important from a practical point of view. Yavari et al. [87] put forward three reasons for the different aging behavior of freestanding thin films and TFC membranes. First, the use of polymer supports and membrane formation procedures may influence segmental mobility of the thin film and thus its aging behavior. Second, industrial membranes may not be annealed above the T_g_, since the porous support may not sustain the high temperature.

**Figure 6:**
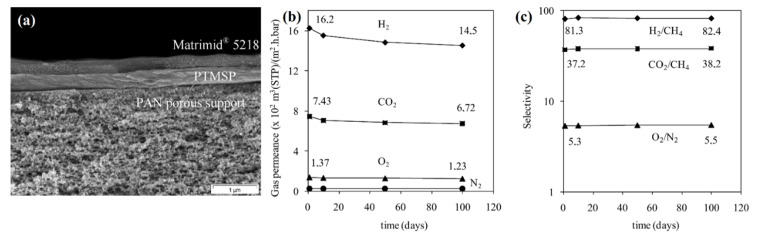
SEM micrograph of a multilayer composite membrane consisting of a cross-linked PTMSP gutter layer and Matrimid^®^ 5218 selective layer coated on PAN/PPS polymeric porous support (**a**) and its long-term performance: (**b**) single gas permeances, measured at 25 °C as a function of time and (**c**) ideal selectivities (adopted from [68]).

Peter and Peinemann [68] fabricated a multi-layer composite membrane with cross-linked PTMSP as the gutter layer (intermediate layer between selective layer and porous PAN-support) with a thickness of 500 nm that enabled the successful deposition of a thin, selective layer made of Matrimid^®^ 5218 (100–500 nm) on top of the gutter layer. The resulting membrane showed attractive, stable long-term performance. The permeances dropped relatively steeply during the first 10 days and tended to level off slowly (Figure 6b). The membranes showed the decline of H_2_, O_2_, CO_2_, N_2_, and CH_4_ permeance of about 19–21% within 100 days with a slight increase in gas permeance (Figure 6c).

Yavari et al. [88] compared the aging behavior of freestanding dense, thin polymer film (400 nm) based on Teflon AF1600, Hyflon AD80, or Hyflon AD40 and TFC membranes with a selective layer made of the same polymer and having a similar thickness deposited on a polyethersulfone ultrafiltration membrane. It was found that the aging rates of both type of membranes turned out to be very similar; for instance, Teflon AF1600 showed the similar tendency in aging in the form of free standing film (400 nm) and a selective layer of TFC membrane (370 nm)—relative N_2_ permeance was 0.89 and 0.86, respectively, after 900 h of storage at 35 °C. A more detailed physicochemical study of the aging of these samples is presented in the subsequent work of Yavari et al. [89], where the glass transition temperatures of the selective layer were measured in situ by nanothermal analysis. It is shown that with a decrease in the thickness of the selective layer, its glass transition temperature decreases; however, after several hundred hours of aging, the glass transition temperature for thinner samples can become higher due to their accelerated aging.

Stabilization of the gas transport properties of TFC membranes based on PTMSP by introducing particles of PAF (30–250 nm) and porous activated carbon (>1000 nm) was studied for the first time by Volkov and coworkers [60,62,64,65,78,79]. Studies have shown that the effectiveness of additives for mitigating physical aging decreases with decreasing thickness of the selective layer, which can be explained by a too-large particle size in a thin selective layer of composite membranes. The development of these membranes was aimed at solving the problems of removing CO_2_ from flue gases. For post-combustion carbon capture, it was shown that the gas separation method could be effectively applied for treatment of a large amount of flue gas if the membrane possesses CO_2_ permeance above 1000 GPU and CO_2_/N_2_ selectivity above 20 [89]. However, in the development of the composite gas separation membranes for post-combustion CO_2_ capture, little attention has been focused on the optimization of the membrane supports that satisfy the conditions of this technology. With this in mind, Bazhenov et al. [62] developed a PTMSP-based TFC membrane suitable as a highly permeable and solvent-resistant nonporous support due to a PTMSP-based gutter layer applied on top of microfilter MFFK-1. A cross-linked PTMSP-based layer of 1–2.5 µm thick was achieved via the semi-interpenetrating network of PTMSP in the cross-linked PEI. The TFC membrane-support demonstrated the CO_2_ permeance of about 20,000 GPUs and ideal CO_2_/N_2_ selectivity of 3.6–3.7.

Table 3 shows comparative aging data for TFC membranes based on high permeability glassy polymers. It can be seen that a smaller relative decrease in gas permeability (81%) was achieved for a composite membrane based on PIM-1 by Bhavsar et al. during a 90-day aging study [90]. However, PTMSP-based membranes with a high PAF-11 content, namely “PTMSP + PEI + 30% PAF-11”, seem preferable due to the nearly twofold permeance of 17,500 GPU (43% of 46,500 GPU) after 450 days of aging [79] compared to 9300 GPU (81% of 11,500 GPU) after 100 days of aging [90].

**Figure 7:**
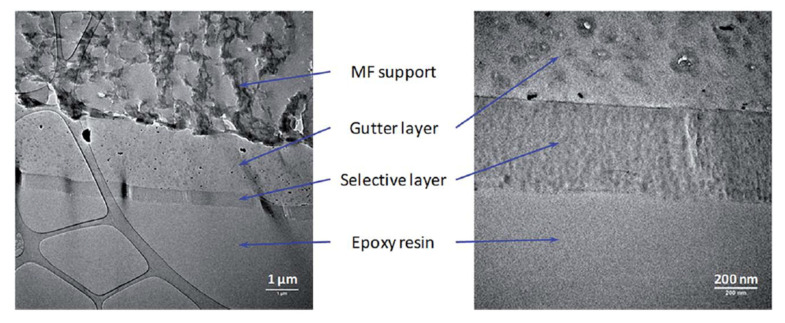
TEM visualization of a composite membrane cross-section. Reproduced from [78] with permission from the Royal Society of Chemistry.

By using the non-porous support developed by Bazhenov et al. [62], the TFC membranes, comprising a thin layer (0.29–0.42 mm) of PIM-1 atop a cross-linked PTMSP/PEI gutter layer (2.07–3.44 mm) on a porous backing material (microfilter MFFK-1), were fabricated by coating PIM-1 solution on the cross-linked PTMSP/PEI support [78]. A high CO_2_ permeance and superior CO_2_/N_2_ selectivity due to a strong synergistic effect were achieved. Figure 7 shows a TEM visualization of a cross-section of a composite membrane. The thicknesses of the PIM-1 and PTMSP/x-PEI layers varied within 200–300 nm and 2.0–3.5 µm, respectively. All samples of composite membranes, which were obtained using solutions of PIM-1 in a mixture of chloroform/trichlorethylene (1:1), demonstrated a significant synergistic increase in CO_2_/N_2_ selectivity (α = 35.8–55.7) compared to PIM-1 (α = 18.5) and cross-linked PTMSP/x-PEI (α = 3.7). It is important that this effect is not observed for PIM-1 solutions in chloroform.

It has been shown that the gas transport properties of composite membranes with a thin PTMSP-based selective layer, namely a semi-interpenetrating network of PTMSP in the cross-linked PEI, can be additionally stabilized owing to the introduction of 10 wt% of PAF-11 nanoparticles [65], or 20 and 30 wt% of PAF-11 nanoparticles [79]. For example, the addition of 30 wt% of PAF-11 nanoparticles preserved the transport characteristics of TFC membrane at 43% (17,600 GPU after 450 days) of its initial values (Table 3).

**Figure 8:**
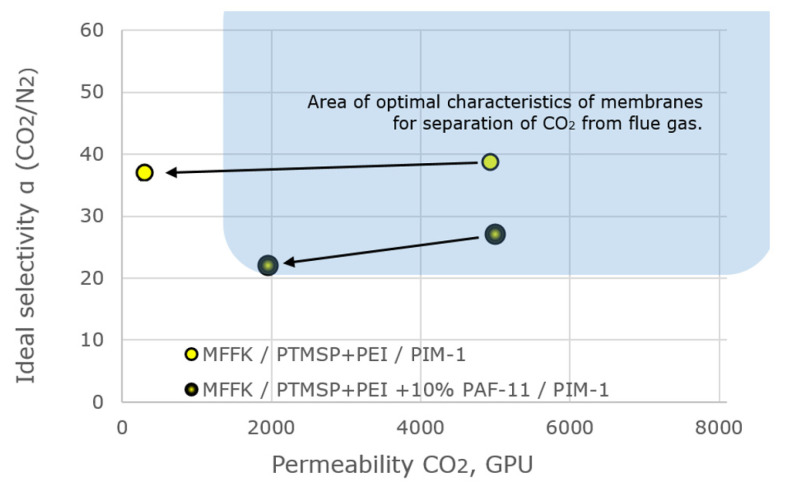
Gas transport characteristics of the developed bilayer PTMSP/PIM thin film composite membranes and their change during the 95-day aging process (shown by arrows) (adopted from [94]).

Using thin layers of the semi-interpenetrating network of PTMSP in the cross-linked PEI with 10% PAF-11 filler as the gutter layer, a new bilayer membrane with a thin layer of PIM-1 on the surface was obtained [94]. The resulting membrane also demonstrated a strong synergistic effect of CO_2_/N_2_ selectivity. Changes in the gas transport characteristics of the bilayer membrane MFFK/PTMSP + PEI/PIM [79] and the bilayer membrane with 10% PAF-11 in gutter layer MFFK/PTMSP + PEI + PAF/PIM [94] are presented in Table 4. It can be seen from the table that the addition of PAF-11 into the gutter layer significantly improved the stability of the bilayer TFC membrane, providing CO_2_ permeance of 1970 GPU after 95 days of aging, compared to 297 GPU for the bilayer membrane without PAF-11 additive. While the selectivity of composite membranes for the CO_2_/N_2_ pair decreases during aging to 20.5, nevertheless, the permeance-selectivity properties of the bilayer TFC membrane with PAF-11 additives still satisfy the demands of the optimal membrane characteristics for post-combustion CO2 capture [89] (Figure 8).

The properties of the composite bilayer membranes with the addition of 10% wt. PAF-11 and without it are plotted on a graph showing the optimal transport and separation properties of membranes for the task of removal of carbon dioxide from flue gases (Figure 8).

PAF-like materials can be considered promising additives for the reduction of physical aging in high free volume glassy polymers; however, their synthesis is quite complex. In this regard, highly porous carbon materials and cross-linked polymers are also being studied as additives in the selective layers of TFC membranes [55,76,77,95]. One of the promising porous additives is highly porous activated carbon material based on infrared pyrolyzed PAN (IR-PAN). According to N_2_ adsorption measurements, the apparent BET surface area and pore volume of PAF-11 are equal to 240 m^2^/g and 0.35 cm^3^/g, respectively [96]. At the same time, the apparent BET surface area and pore volume of IR-PAN were equal to 2450 m^2^/g and 1.06 cm^3^/g, respectively [97].

**Figure 9:**
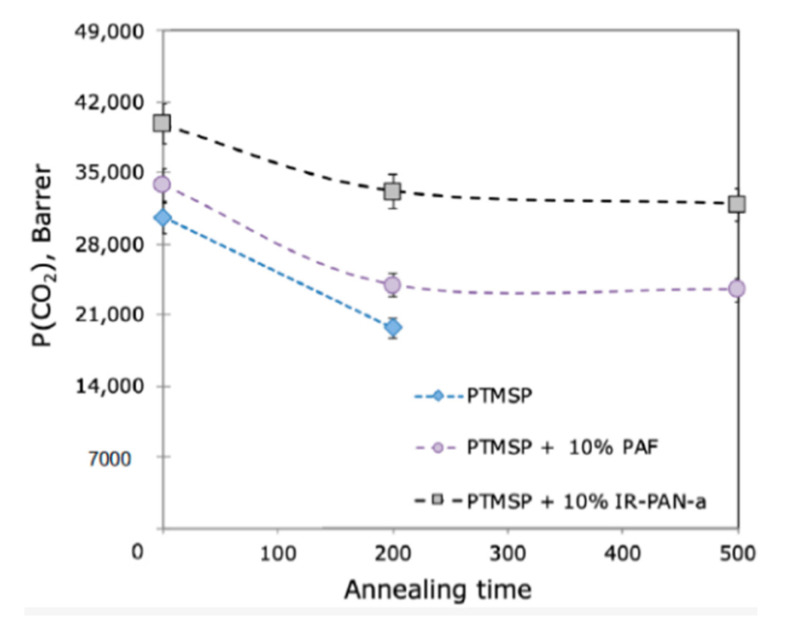
Comparison of the effect of adding IR-PAN and PAF-11 on the properties of homogeneous membranes (35–40 µm) based on PTMSP: dependence of the CO_2_ permeability coefficient on the membrane annealing time at 100 °C in air (adopted from [60]).

Bakhtin et al. [60] compared the effectiveness of IR-PAN filler with PAF-11. Figure 9 shows the dependence of the CO_2_ permeability coefficient of PTMSP hybrid homogeneous membranes (35–40 µm) with additions of IR-PAN and PAF-11 on the annealing time at 100 °C. As can be seen from the figure, the addition of IR-PAN leads to an increase in the CO_2_ permeability coefficient of the PTMSP/IR-PAN hybrid membrane compared to the PTMSP/PAF-11 one. In addition, the relative drop in CO_2_ permeability after 500 h of annealing at 100 °C for a sample with 10 wt% PAF-11 was 30%, while the addition of IR-PAN reduces this drop to 20%. The aging of TFC membranes with a PTMSP + PEI + IR-PAN selective layer (0.8–1.8 µm thick) was also studied. TFC membrane permeance was 6300 GPU (30% of initial permeance) after 11,000 h of aging at ambient laboratory conditions. A further increase in the content of the additive (20 and 30 wt%) did not significantly affect the properties of the membranes.

## 4. Conclusions

To date, membranes based on glassy polymers (polysulfone, cellulose acetate, polyimide, substituted polycarbonate, and polyphenylene oxide) are commonly used in gas separation processes, such as acid gas treatment or nitrogen generation from air. However, glassy polymers undergo a physical aging process resulting in significant reduction of membrane fluxes over time.

Several techniques to mitigate the physical aging of glassy polymers, such as post-modification of prepared membranes via crosslinking, addition of porous and nonporous nanomaterials, and the combination of these two approaches have been considered. Membrane material behavior has been discussed with respect to the dense membrane’s gas transport properties. The rate of glassy polymer aging depends greatly on its thermal history, aging temperature, and the initial thickness of its film.

Specific emphasis is placed on the physical aging of thin film composite membranes and the stabilization of their gas transport properties. In the case of thin-film composite membranes, the same dependence of the aging rate on the selective layer thickess is also observed. However, it is possible to choose a membrane composition that will significantly reduce the polymer aging, which was demonstrated by the example of a two-layer TFC membrane based on Matrimid^®^ and cross-linked PTMSP as a gutter layer.

## Figures and Tables

**Table 3:** Comparison of the TFC membranes aging.

Selective Layer	Selective Layer Thickness, µm	CO_2_ Permeance of Fresh As-Cast Membrane, GPU	Aging Time (Ambient Conditions)	CO_2_ Permeance Q/Q_0_, %	CO_2_/N_2_ Ideal Selectivity α/α_0_, %	Reference
PTMSP+10% PAF-11	1.7	1700	>600 days	8.5	314	[64]
	6.8	6500		5.5	137	
PIM-1/C-HCP	2.0	22,000	100 days	37	150	[90]
PIM-1	0.3	8000	90 days	3.7	96	[78]
PIM-1	0.7	4300	56 days	11	166	[91]
PIM-1/MOF-74-Ni		5000		24	90	
PIM-1/NH2-UiO-66		7500		12	100	
PTMSP + PEI	1.2	15,100	>425 days	23	139	[60]
PTMSP + PEI + 10% IR-PAN-a		23,700		17	158	
PTMSP + PEI + 20% IR-PAN-a	1.8	24,700		26	133	
PTMSP + PEI + 30% IR-PAN-a		24,100		22	111	
PTMSP + PEI + 10% IR-PAN-aM		20,900		30	107	
PTMSP + PEI + 20% IR-PAN-aM	1.0	24,500		27	110	
PTMSP + PEI + 30% IR-PAN-aM		25,100		27	114	
Carbon molecular sieves (PDMS pyrolysis) precursor	0.087	239	45 days	9.6	50	[92]
Carbon molecular sieves (PDMS pyrolysis) 500 °C	0.069	294		9.9	110	
Carbon molecular sieves (PDMS pyrolysis) 600 °C	0.082	320		0.9	166	
Carbon molecular sieves (PDMS pyrolysis) 700 °C	0.072	8		17.5	250	
PU/PIM-1	30	11	60 days	82	-	[93]
PTMSP	1.0	27,700	450 days	14	-	[79]
PTMSP + PEI	1.1	21,000		30	-	
PTMSP + PEI + 20% PAF-11	1.0	34,400		42	-	
PTMSP + PEI + 30% PAF-11	1.1	40,600		43	-	

**Table 4:** Aging of thin film composite PTMSP/PIM bilayer membranes over time (adopted from [94]).

Membrane	Time Since Formation, Days	Permeanse, GPU		Ideal Selectivity α (CO_2_/N_2_)
		N_2_	CO_2_	
MFFK/gutter layer − PTMSP + cross-linked PEI/PIM-1	0	127	4930	38.8
	94	8	297	37.1
MFFK/gutter layer − PTMSP + cross-linked − PEI + 10% PAF-11/PIM-1	0	185	5000	27.0
	95	96	1970	20.5

## Data Availability

Not applicable.

## References

[1] Galizia M., Chi W.S., Smith Z.P., Merkel T.C., Baker R.W., Freeman B.D. (2017). 50th anniversary perspective: Polymers and mixed matrix membranes for gas and vapor separation: A review and prospective opportunities. Macromolecules.

[2] Yampol’skii Y.P., Volkov V.V. (1991). Studies in gas permeability and membrane gas separation in the Soviet Union. J. Membr. Sci..

[3] Filippov S.P., Yaroslavtsev A.B. (2021). Hydrogen energy: Development prospects and materials. Russ. Chem. Rev..

[4] Baker R.W., Lokhandwala K. (2008). Natural gas processing with membranes: An overview. Ind. Eng. Chem. Res..

[5] Sanders D.F., Smith Z.P., Guo R., Robeson L.M., McGrath J.E., Paul D.R., Freeman B.D. (2013). Energy-efficient polymeric gas separation membranes for a sustainable future: A review. Polymer.

[6] Valappil R.S.K., Ghasem N., Al-Marzouqi M. (2021). Current and future trends in polymer membrane-based gas separation technology: A comprehensive review. J. Ind. Eng. Chem..

[7] Cangialosi D., Boucher V.M., Alegria A., Colmenero J. (2013). Physical aging in polymers and polymer nanocomposites: Recent results and open questions. Soft Matter.

[8] Masuda T., Isobe E., Higashimura T. (1983). Poly [1-(trimethylsilyl)-1-propyne]: A new high polymer synthesized with transition-metal catalysts and characterized by extremely high gas permeability. J. Am. Chem. Soc..

[9] Khotimsky V.S., Tchirkova M.V., Litvinova E.G., Rebrov A.I., Bondarenko G.N. (2003). Poly [1-(trimethylgermyl)-1-propyne] and poly [1-(trimethylsilyl)-1-propyne] with various geometries: Their synthesis and properties. J. Polym. Sci. Part A Polym. Chem..

[10] Shtennikova I.N., Kolbina G.F., Yakimansky A.V., Plate N.A., Khotimsky V.S., Litvinova E.G. (1999). Experimental and theoretical investigation of optical properties of poly-(1-trimethylsilyl-1 propyne) molecules in solution. Eur. Polym. J..

[11] Volkov V.V. (1991). Free volume structure and transport properties of glassy polymers—Materials for separating membranes. Polym. J..

[12] Srinivasan R., Auvil S.R., Burban P.M. (1994). Elucidating the mechanism(s) of gas transport in poly [1-(trimethylsilyl)-1-propyne](PTMSP) membranes. J. Membr. Sci..

[13] Hofmann D., Entrialgo-Castano M., Lerbret A., Heuchel M., Yampolskii Y. (2003). Molecular modeling investigation of free volume distributions in stiff chain polymers with conventional and ultrahigh free volume: Comparison between molecular modeling and positron lifetime studies. Macromolecules.

[14] Consolati G., Genco I., Pegoraro M., Zanderighi L. (1996). Positron annihilation lifetime (PAL) in poly [1-(trimethyl-silyl) propine](PTMSP): Free volume determination and time dependence of permeability. J. Polym. Sci. Part B Polym. Phys..

[15] Ahmad M.Z., Castro-Muñoz R., Budd P.M. (2020). Boosting gas separation performance and suppressing the physical aging of polymers of intrinsic microporosity (PIM-1) by nanomaterial blending. Nanoscale.

[16] Morisato A., He Z., Pinnau I. (1999). Mixed-Gas Properties and Physical Aging of Poly(4-methyl-2-pentyne). Polymer Membranes for Gas and Vapor Separation: Chemistry and Materials Science.

[17] Nagai K., Toy L.G., Freeman B.D., Teraguchi M., Kwak G., Masuda T., Pinnau I. (2002). Gas permeability and n-butane solubility of poly (1-trimethylgermyl-1-propyne). J. Polym. Sci. Part B Polym. Phys..

[18] Wozniak A.I., Bermesheva E.V., Borisov I.L., Volkov A.V., Petukhov D.I., Gavrilova N.N., Shantarovich V.P., Asachenko A.F., Topchiy M.A., Finkelshtein E.S. (2022). Switching on/switching off solubility controlled permeation of hydrocarbons through glassy polynorbornenes by the length of side alkyl groups. J. Membr. Sci..

[19] Bermeshev M.V., Syromolotov A.V., Gringolts M.L., Starannikova L.E., Yampolskii Y.P., Finkelshtein E.S. (2011). Synthesis of high molecular weight poly [3-{tris (trimethylsiloxy) silyl} tricyclononenes-7] and their gas permeation properties. Macromolecules.

[20] Merkel T.C., Pinnau I., Prabhakar R., Freeman B.D., Yampolskii Y., Pinnau I., Freeman B.D. (2006). Gas and vapor transport properties of perfluoropolymers. Materials Science of Membranes for Gas and Vapor Separation.

[21] Pinnau I., Toy L.G. (1996). Gas and vapor transport properties of amorphous perfluorinated copolymer membranes based on 2, 2-bistrifluoromethyl-4, 5-difluoro-1, 3-dioxole/tetrafluoroethylene. J. Membr. Sci..

[22] Alentiev A.Y., Yampolskii Y.P., Shantarovich V.P., Nemser S.M., Plate N.A. (1997). High transport parameters and free volume of perfluorodioxole copolymers. J. Membr. Sci..

[23] Neki K., Geil P.H. (1973). Morphology-property studies of amorphous polycarbonate. J. Macromol. Sci. Part B Phys..

[24] Yeh G.S.Y. (1973). Yielding of glassy polymer on a microstructural level. J. Macromol. Sci. Part B Phys..

[25] Huang Y., Paul D.R. (2006). Physical aging of thin glassy polymer films monitored by optical properties. Macromolecules.

[26] McCaig M.S., Paul D.R. (2000). Effect of film thickness on the changes in gas permeability of a glassy polyarylate due to physical aging Part I. Experimental observations. Polymer.

[27] Rowe B.W., Freeman B.D., Paul D.R. (2009). Physical aging of ultrathin glassy polymer films tracked by gas permeability. Polymer.

[28] McCaig M.S., Paul D.R., Barlow J.W. (2000). Effect of film thickness on the changes in gas permeability of a glassy polyarylate due to physical aging Part II. Mathematical model. Polymer.

[29] Huang Y., Paul D.R. (2004). Physical aging of thin glassy polymer films monitored by gas permeability. Polymer.

[30] Kelman S.D., Rowe B.W., Bielawski C.W., Pas S.J., Hill A.J., Paul D.R., Freeman B.D. (2008). Crosslinking poly [1-(trimethylsilyl)-1-propyne] and its effect on physical stability. J. Membr. Sci..

[31] Horn N.R., Paul D.R. (2011). Carbon dioxide plasticization of thin glassy polymer films. Polymer.

[32] Horn N.R., Paul D.R. (2012). Carbon dioxide sorption and plasticization of thin glassy polymer films tracked by optical methods. Macromolecules.

[33] Tiwari R.R., Smith Z.P., Lin H., Freeman B.D., Paul D.R. (2014). Gas permeation in thin films of “high free-volume” glassy perfluoropolymers: Part I. Physical aging. Polymer.

[34] Wang H., Chung T.-S., Paul D.R. (2014). Physical aging and plasticization of thick and thin films of the thermally rearranged ortho-functional polyimide 6FDA−HAB. J. Membr. Sci..

[35] Rowe B.W., Pas S.J., Hill A.J., Suzuki R., Freeman B.D., Paul D. (2009). A variable energy positron annihilation lifetime spectroscopy study of physical aging in thin glassy polymer films. Polymer.

[36] Tiwari R.R., Jin J.Y., Freeman B.D., Paul D.R. (2017). Physical aging, CO_2_ sorption and plasticization in thin films of polymer with intrinsic microporosity (PIM-1). J. Membr. Sci..

[37] Merrick M.M., Sujanani R., Freeman B.D. (2020). Glassy polymers: Historical findings, membrane applications, and unresolved questions regarding physical aging. Polymer.

[38] Low Z.X., Budd P.M., McKeown N.B., Patterson D.A. (2018). Gas permeation properties, physical aging, and its mitigation in high free volume glassy polymers. Chem. Rev..

[39] Lau C.H., Konstas K., Thornton A.W., Liu A.C.Y., Mudie S., Kennedy D.F., Shaun C., Howard S.C., Hill A.J., Hill M.R. (2015). Gas-separation membranes loaded with porous aromatic frameworks that improve with age. Angew. Chem..

[40] Kamble A.R., Patel C.M., Murthy Z.V.P. (2021). A review on the recent advances in mixed matrix membranes for gas separation processes. Renew. Sustain. Energy Rev..

[41] Wang Y., Ma X., Ghanem B.S., Alghunaimi F., Pinnau I., Han Y. (2018). Polymers of intrinsic microporosity for energy-intensive membrane-based gas separations. Mater. Today Nano.

[42] Dai Z., Ansaloni L., Deng L. (2016). Recent advances in multi-layer composite polymeric membranes for CO_2_ separation: A review. Green Energy Environ..

[43] León N.E., Liu Z., Irani M., Koros W.J. (2022). How to get the best gas separation membranes from state-of-the-art glassy polymers. Macromolecules.

[44] Yampol’skii Y.P., Shishatskii S.M., Shantorovich V.P., Antipov E.M., Kuzmin N.N., Rykov S.V., Plate N.A. (1993). Transport characteristics and other physicochemical properties of aged poly (1-(trimethylsilyl)-1-propyne). J. Appl. Polym. Sci..

[45] Struik L.C.E. (1978). Physical Aging in Amorphous Glassy Polymers and Other Materials.

[46] Pfromm P.H., Koros W.J. (1995). Accelerated physical ageing of thin glassy polymer films: Evidence from gas transport measurements. Polymer.

[47] Dorkenoo K.D., Pfromm P.H. (1999). Experimental evidence and theoretical analysis of physical aging in thin and thick amorphous glassy polymer films. J. Polym. Sci. Part B Polym. Phys..

[48] Forrest J.A., Dalnoki-Veress K. (2001). The glass transition in thin polymer films. Adv. Colloid Interface Sci..

[49] Forrest J.A. (2002). A decade of dynamics in thin films of polystyrene: Where are we now?. Eur. Phys. J. E.

[50] Huang Y., Paul D.R. (2007). Effect of film thickness on the gas-permeation characteristics of glassy polymer membranes. Ind. Eng. Chem. Res..

[51] Shishatskii A.M., Yampol’skii Y.P., Peinemann K.V. (1996). Effects of film thickness on density and gas permeation parameters of glassy polymers. J. Membr. Sci..

[52] Pfromm P.H., Yampolskii Y., Pinnau I., Freeman B.D. (2006). The impact of physical aging of amorphous glassy polymers on gas separation membranes. Materials Science of Membranes for Gas and Vapor Separation.

[53] Castro-Munoz R., Fila V., Dung C.T. (2017). Mixed matrix membranes based on PIMs for gas permeation: Principles, synthesis, and current status. Chem. Eng. Commun..

[54] Olivieri L., Ligi S., De Angelis M.G., Cucca G., Pettinau A. (2015). Effect of graphene and graphene oxide nanoplatelets on the gas permselectivity and aging behavior of poly (trimethylsilyl propyne)(PTMSP). Ind. Eng. Chem. Res..

[55] Lau C.H., Nguyen P.T., Hill M.R., Thornton A.W., Konstas K., Doherty C.M., Mulder R.J., Bourgeois L., Liu A.C.Y., Sprouster D.J. (2014). Ending Aging in Super Glassy Polymer Membranes. Angew. Chem..

[56] Lau C.H., Konstas K., Doherty C.M., Kanehashi S., Ozcelik B., Kentish S.E., Hill M.R. (2015). Tailoring Physical Aging in Super Glassy Polymers with Functionalized Porous Aromatic Frameworks for CO_2_ Capture. Chem. Mater..

[57] Kitchin M., Teo J., Konstas K., Lau C.H., Sumby C.J., Thornton A.W., Doonan C.J., Hill M.R. (2015). AIMs: A new strategy to control physical aging and gas transport in mixed-matrix membranes. J. Mater. Chem. A.

[58] Lau C.H., Mulet X., Konstas K., Doherty C.M., Sani M.A., Separovic F., Hill M.R., Wood C.D. (2016). Hypercrosslinked Additives for Ageless Gas-Separation Membranes. Angew. Chem. Int. Ed..

[59] Liu J., Xiao Y., Liao K.S., Chung T.S. (2017). Highly permeable and aging resistant 3D architecture from polymers of intrinsic microporosity incorporated with beta-cyclodextrin. J. Membr. Sci..

[60] Bakhtin D., Bazhenov S., Polevaya V., Grushevenko E., Makaev S., Karpacheva G., Volkov A. (2020). Aging of Thin-Film Composite Membranes Based on Crosslinked PTMSP/PEI Loaded with Highly Porous Carbon Nanoparticles of Infrared Pyrolyzed Polyacrylonitrile. Membranes.

[61] Jia J., Baker G.L. (1998). Cross-linking of poly [1-(trimethylsilyl)-1-propyne] membranes using bis (aryl azides). J. Polym. Sci. B Polym. Phys..

[62] Bazhenov S.D., Borisov I.L., Bakhtin D.S., Rybakova A.N., Khotimskiy V.S., Molchanov S.P., Volkov V.V. (2016). High-permeance crosslinked PTMSP thin-film composite membranes as supports for CO_2_ selective layer formation. Green Energy Environ..

[63] Shao L., Samseth J., Hägg M.B. (2009). Crosslinking and stabilization of nanoparticle filled poly (1-trimethylsilyl-1-propyne) nanocomposite membranes for gas separations. J. Appl. Polym. Sci..

[64] Bakhtin D.S., Kulikov L.A., Legkov S.A., Khotimskiy V.S., Levin I.S., Borisov I.L., Maksimov A.L., Volkov V.V., Karakhanov E.A., Volkov A.V. (2018). Aging of thin-film composite membranes based on PTMSP loaded with porous aromatic frameworks. J. Membr. Sci..

[65] Bakhtin D.S., Kulikov L.A., Bondarenko G.N., Vasilevskii V.P., Maksimov A.L., Volkov A.V. (2018). Stabilization of Gas Transport Properties of Composite Membranes with a Thin PTMSP Selective Layer by Adding Porous Aromatic Framework Nanoparticles and Simultaneous Polymer Crosslinking. Pet. Chem..

[66] Bakhtin D.S., Kulikov L.A., Maksimov A.L., Volkov A.V. (2020). Composite Membranes Based on the Poly(1-trimethylsylyl-1-propine): Influence of the Porous Aromatic Frameworks Produced from the Friedel-Crafts Reaction and Introduced into the Polymer Matrix. Russ. J. Appl. Chem..

[67] Bakhtin D.S., Malakhov A.O., Polevaya V.G., Kulikov L.A., Grekhov A.M., Bazhenov S.D., Volkov A.V. (2021). Behavior of Polytrimethylsilylpropyne-Based Composite Membranes in the Course of Continuous and Intermittent Gas Permeability Measurements. Russ. J. Appl. Chem..

[68] Peter J., Peinemann K.V. (2009). Multilayer composite membranes for gas separation based on crosslinked PTMSP gutter layer and partially crosslinked Matrimid^®^ 5218 selective layer. J. Membr. Sci..

[69] Shao L., Samseth J., Hägg M.B. (2009). Crosslinking and stabilization of nanoparticle filled PMP nanocomposite membranes for gas separations. J. Membr. Sci..

[70] Fritsch D., Merten P., Heinrich K., Lazar M., Priske M. (2012). High performance organic solvent nanofiltration membranes: Development and thorough testing of thin film composite membranes made of polymers of intrinsic microporosity (PIMs). J. Membr. Sci..

[71] Hou R., Ghanem B.S., Smith S.J., Doherty C.M., Setter C., Wang H., Pinnau I., Hill M.R. (2020). Highly permeable and selective mixed-matrix membranes for hydrogen separation containing PAF-1. J. Mater. Chem. A.

[72] Lau C.H., Konstas K., Doherty C.M., Smith S.J., Hou R., Wang H., Carta M., Yoon H., Park J., Freeman B.D. (2020). Tailoring molecular interactions between microporous polymers in high performance mixed matrix membranes for gas separations. Nanoscale.

[73] Ben T., Ren H., Ma S., Cao D., Lan J., Jing X., Wang W., Xu J., Deng F., Simmons J.M. (2009). Targeted synthesis of a porous aromatic framework with high stability and exceptionally high surface area. Angew. Chem..

[74] Cheng X.Q., Konstas K., Doherty C.M., Wood C.D., Mulet X., Xie Z., Ng D., Hill M.R., Shao L., Lau C.H. (2017). Hyper-Cross-Linked Additives that Impede Aging and Enhance Permeability in Thin Polyacetylene Films for Organic Solvent Nanofiltration. ACS Appl. Mater. Interfaces.

[75] Smith S.J.D., Hou R., Konstas K., Akram A., Lau C.H., Hill M.R. (2020). Control of Physical Aging in Super-Glassy Polymer Mixed Matrix Membranes. Acc. Chem. Res..

[76] Golubev G.S., Borisov I.L., Litvinova E.G., Khotimsky V.S., Bakhtin D.S., Pastukhov A., Davankov V.A., Volkov V.V. (2017). A novel hybrid material based on polytrimethylsilylpropyne and hypercrosslinked polystyrene for membrane gas separation and thermopervaporation. Pet. Chem..

[77] Volkov A.V., Bakhtin D.S., Kulikov L.A., Terenina M.V., Golubev G.S., Bondarenko G.N., Legkov S.A., Shandryuk G.A., Volkov V.V., Khotimskiy V.S. (2016). Stabilization of gas transport properties of PTMSP with porous aromatic framework: Effect of annealing. J. Membr. Sci..

[78] Borisov I., Bakhtin D., Luque-Alled J.M., Rybakova A., Makarova V., Foster A.B., Volkov V., Volkov A. (2019). Synergistic enhancement of gas selectivity in thin film composite membranes of PIM-1. J. Mater. Chem. A..

[79] Bakhtin D.S., Malakhov A.O., Volkov A.V., Kulikov L.A., Petrova I.V., Borisov I.L., Bazhenov S.D. (2023). Mitigating of Thin-Film Composite PTMSP Membrane Aging by Introduction of Porous Rigid and Soft Branched Polymeric Additives. Membranes.

[80] Merkel T.C., Freeman B.D., Spontak R.J., He Z., Pinnau I., Meakin P., Hill A.J. (2003). Sorption, Transport, and Structural Evidence for Enhanced Free Volume in Poly(4-methyl-2-pentyne)/Fumed Silica Nanocomposite Membranes. Chem. Mater..

[81] Hill A.J., Pas S.J., Bastow T.J., Burgar M.I., Nagai K., Toy L.G., Freeman B.D. (2004). Influence of methanol conditioning and physical aging on carbon spin-lattice relaxation times of poly(1-trimethylsilyl-1-propyne). J. Membr. Sci..

[82] Volkov A.V., Tsarkov S.E., Gokzhaev M.B., Bondarenko G.N., Legkov S.A., Kukushkina Y.A., Volkov V.V. (2012). Nanofiltration and sorption of organic solvents in poly(1-trimethylsilyl-1-propyne) samples of different microstructures. Petrol. Chem..

[83] Yu M., Foster A.B., Scholes C.A., Kentish S.E., Budd P.M. (2023). Methanol Vapor Retards Aging of PIM-1 Thin Film Composite Membranes in Storage. ACS Macro Lett..

[84] Abdulhamid M.A., Lai H.W., Wang Y., Jin Z., Teo Y.C., Ma X., Pinnau I., Xia Y. (2019). Microporous polyimides from ladder diamines synthesized by facile catalytic arene–norbornene annulation as high-performance membranes for gas separation. Chem. Mater..

[85] Ma X., Lai H.W., Wang Y., Alhazmi A., Xia Y., Pinnau I. (2020). Facile Synthesis and Study of Microporous Catalytic Arene-Norbornene Annulation–Tröger’s Base Ladder Polymers for Membrane Air Separation. ACS Macro Lett..

[86] Lai H.W., Benedetti F.M., Ahn J.M., Robinson A.M., Wang Y., Pinnau I., Xia Y. (2022). Hydrocarbon ladder polymers with ultrahigh permselectivity for membrane gas separations. Science.

[87] Yavari M., Le T., Lin H. (2017). Physical aging of glassy perfluoropolymers in thin film composite membranes. Part I. Gas transport properties. J. Membr. Sci..

[88] Yavari M., Maruf S., Ding Y., Lin H. (2017). Physical aging of glassy perfluoropolymers in thin film composite membranes. Part II. Glass transition temperature and the free volume model. J. Membr. Sci..

[89] Merkel T.C., Lin H., Wei X., Baker R. (2010). Power plant post-combustion carbon dioxide capture: An opportunity for membranes. J. Membr. Sci..

[90] Bhavsar R.S., Mitra T., Adams D.J., Cooper A.I., Budd P.M. (2018). Ultrahigh-permeance PIM-1 based thin film nanocomposite membranes on PAN supports for CO_2_ separation. J. Membr. Sci..

[91] Liu M., Nothling M.D., Webley P.A., Jin J., Fu Q., Qiao G.G. (2020). High-throughput CO_2_ capture using PIM-1@ MOF based thin film composite membranes. Chem. Eng. J..

[92] Ogieglo W., Puspasari T., Ma X., Pinnau I. (2020). Sub-100 nm carbon molecular sieve membranes from a polymer of intrinsic microporosity precursor: Physical aging and near-equilibrium gas separation properties. J. Membr. Sci..

[93] Fan S.T., Tan M., Liu W.T., Li B.J., Zhang S. (2022). MOF-layer composite polyurethane membrane increasing both selectivity and permeability: Pushing commercial rubbery polymer membranes to be attractive for CO_2_ separation. Sep. Purif. Technol..

[94] Bakhtin D.S. (2023). Development of Gas Separation Membranes Based on PTMSP with Enhanced Stability of Characteristics in Time. Ph.D. Thesis.

[95] Mitra T., Bhavsar R.S., Adams D.J., Budd P.M., Cooper A.I. (2016). PIM-1 mixed matrix membranes for gas separations using cost-effective hypercrosslinked nanoparticle fillers. Chem. Commun..

[96] Kulikov L.A., Bakhtin D.S., Polevaya V.G., Balynin A.V., Maksimov A.L., Volkov A.V. (2019). Friedel-Crafts Synthesis of New Porous Aromatic Frameworks for Stabilizing Gas Transport Properties of Highly Permeable Glassy Polymers. Russ. J. Appl. Chem..

[97] Efimov M.N., Vasilev A.A., Muratov D.G., Baranchikov A.E., Karpacheva G.P. (2019). IR radiation assisted preparation of KOH-activated polymer-derived carbon for methylene blue adsorption. J. Environ. Chem. Eng..

